# Shape Memory Polymer Foams With Phenolic Acid-Based Antioxidant and Antimicrobial Properties for Traumatic Wound Healing

**DOI:** 10.3389/fbioe.2022.809361

**Published:** 2022-02-17

**Authors:** Changling Du, Jingyi Liu, David Anthony Fikhman, Katheryn Shi Dong, Mary Beth Browning Monroe

**Affiliations:** Department of Biomedical and Chemical Engineering and Syracuse BioInspired Institute, Syracuse University, Syracuse, NY, United States

**Keywords:** phenolic acid, polyurethane, shape memory polymer, antimicrobial, antioxidant, hemostatic dressing

## Abstract

The leading cause of trauma-related death before arrival at a hospital is uncontrolled blood loss. Upon arrival at the hospital, microbial infections in traumatic wounds become an additional factor that increases mortality. The development of hemostatic materials with antimicrobial and antioxidant properties could improve morbidity and mortality in these wounds. To that end, phenolic acids (PAs) were successfully incorporated into the network of shape memory polymer (SMP) polyurethane foams by reacting them with isocyanates. Resulting PA-containing SMP foam shape memory properties, antimicrobial and antioxidant activity, and blood and cell interactions were characterized. Results showed that p-coumaric, vanillic, and ferulic acids were successfully incorporated into the SMP foams. The PA-containing SMP foams retained the antimicrobial and antioxidant properties of the incorporated PAs, with ∼20% H_2_O_2_ scavenging and excellent antimicrobial properties again *E. coli* (∼5X reduction in CFUs vs. control foams), *S. aureus* (∼4.5X reduction in CFUs vs. control foams, with comparable CFU counts to clinical control), and *S. epidermidis* (∼25–120X reduction in CFUs vs. control foams, with comparable CFU counts to clinical control). Additionally, appropriate thermal and shape memory properties of PA foams could enable stable storage in low-profile secondary geometries at temperatures up to ∼55°C and rapid expand within ∼2 min after exposure to water in body temperature blood. PA foams had high cytocompatibility (>80%), non-hemolytic properties, and platelet attachment and activation, with improved cytocompatibility and hemocompatibility in comparison with clinical, silver-based controls. The incorporation of PAs provides a natural non-antibiotic approach to antimicrobial SMP foams with antioxidant properties. This system could improve outcomes in traumatic wounds to potentially reduce bleeding-related deaths and subsequent infections.

## Introduction

On the battlefield, uncontrolled bleeding is the primary cause of trauma-related mortality. Of these deaths, 33–56% occur during the prehospital period. (Kauvar et al., 2006). Furthermore, polymicrobial infections occur in 39% of traumatic wounds within the first week after injury, complicating healing processes in patients who do survive. (Heckbert 1998). The primary current treatment for hemorrhage control includes gauze that has been modified with anticoagulants. QuikClot^®^ Combat Gauze (QCCG) is widely used on the battlefield and in hospitals, but its prolonged implantation increases risk of embolism. According to a pig study conducted by Otrocka-Domagala et al., when QCCG was left in the wound for more than 24 h, fibrin-gaseous embolic material was found in the pulmonary arteries. (Otrocka-Domagała 2016). Newer clinically-available approaches to bleeding control include XStat™ (RevMedx, lnc., Wilsonville, OR), which is comprised of a syringe-like applicator loaded with multiple small compressed cellulose sponges that can be injected into a deep wound, where they expand rapidly. (Mueller et al., 2012). XStat has short hemostasis times, portability, and ease of use. However, XStat does not protect against infection, and its mean removal time is ∼22 times longer than that of gauze, because individual sponges need to be removed from the wound bed. (Kragh et al., 2015). This process could cause secondary injury to the wound and further increase infection risks.

In current research, a variety of biomaterials have been explored for use as hemostatic agents such as fibrin (Reiss and Oz 1996), chitosan (Ong et al., 2008) or cryogels (Zhao et al., 2018), but no options have emerged that effectively reduce bleeding deaths and subsequent complications, such as wound infection. Some approaches to this issue include poly (ethylene glycol)/starch-based hydrogels with antimicrobial peptides incorporated into the backbone. (Yang et al., 2019). These materials demonstrate clotting efficacy *in vitro* and *in vivo* and antimicrobial properties. However, the cost and time associated with solid-phase peptide synthesis may limit the scale up of these materials, and they do not have a clear mechanism for easy implantation into deep, irregularly-shaped traumatic wounds. An alternative approach includes an injectable hydrogel based on oxidized dextran with antimicrobial and readily available *ε*-poly-L-lysine and fibroblast growth factor. (Gao et al., 2020). These materials have promising antimicrobial and hemostatic properties, but their storage stability in remote field care conditions may be limited and the ability to stably apply these very soft materials to large hemorrhagic wounds is questionable. Despite these and other promising approaches, an antimicrobial hemostatic material that is easy to apply and effective at promoting clotting is still required to improve traumatic wound treatment outcomes.

To address this need, thermally induced polyurethane shape memory polymer (SMP) foams provide a promising option. In previous work, a highly crosslinked thermoset polyurethane SMP foam system was developed. (Singhal et al., 2014). These SMP foams are a class of porous smart materials that can be programmed into a low-profile secondary shape by heating, compressing, and cooling. Under dry conditions up to ∼50°C, these foams maintain their compressed shape for easy storage and delivery into wounds. When the foams are placed into a wound and exposed to water in body temperature blood, they recover and expand to their primary shape to fill the wound and induce clotting. SMP foams were previously implanted in a porcine aneurysm model, where they showed excellent biocompatibility, complete healing, and minimal inflammatory response after 90 days*.* (Rodriguez et al., 2014; Boyle et al., 2016). In more recent work, these SMP foams demonstrated improved survival compared to clinical controls in a lethal liver injury in pigs. (Beaman 2021). This previous work indicates that these polyurethane SMP foams are a promising hemostatic material for trauma-related wounds due to their shape memory properties, which enable easy application; biocompatibility; and hemostatic abilities.

An additional benefit of these materials is their high synthetic tunability, enabling incorporation of desired functional groups that could aid in healing; the goal in this work is to provide antimicrobial SMP foams to reduce infection risks. To reduce complications associated with drug-resistant infections, natural antimicrobials are increasingly pursued for clinical use. Honey has been reported to promote wound healing and reduce microbial infection since the 19th century. (Efem 1988; Molan and Betts 2004). For example, Revamil^®^ and Manuka honey are medical-grade honeys for clinical applications. Both inhibit bacterial growth, including methicillin-resistant *Staphylococcus aureus.* (Kwakman et al., 2010). The antibacterial components that have been identified in honey include several phenolic compounds, among others. These phenolic species originating from plant nectar provide non-peroxide antibacterial activity. (Kwakman and Zaat 2012). Specifically, phenolic acids (PAs) have demonstrated excellent antimicrobial activity in several studies and have effectiveness against multidrug resistant organisms (MDROs). (Wahdan 1998; Nascimento et al., 2000; Merkl 2010).

In addition to their antimicrobial properties, PAs contain hydrogen donor groups, such as hydroxyl and methoxy groups, that can react with oxidants, forming resonance-stabilized phenoxy radicals to reduce reactive oxygen species (ROS) concentrations. (Natella et al., 1999; Oldham et al., 2002; Sroka and Cisowski 2003; Merkl et al., 2010). ROS are essential for native wound healing by aiding in infection control, acting as secondary messengers for immune cells, and regulating angiogenesis. (Dunnill et al., 2017). However, prolonged exposure of wounds to high ROS levels can cause cellular damage and inhibit healing. Therefore, the use of antioxidants to remove ROS during healing can interrupt the chronic inflammatory cycle and aid in healing. Beyond their antimicrobial and antioxidant properties, some PAs, including p-coumaric acid and vanillic acid, have also been shown to promote blood coagulation activity. (Huang et al., 2010). Thus, PAs have the potential to confer antimicrobial and antioxidant properties to hemostatic agents while aiding in coagulation.

**FIGURE 1:**
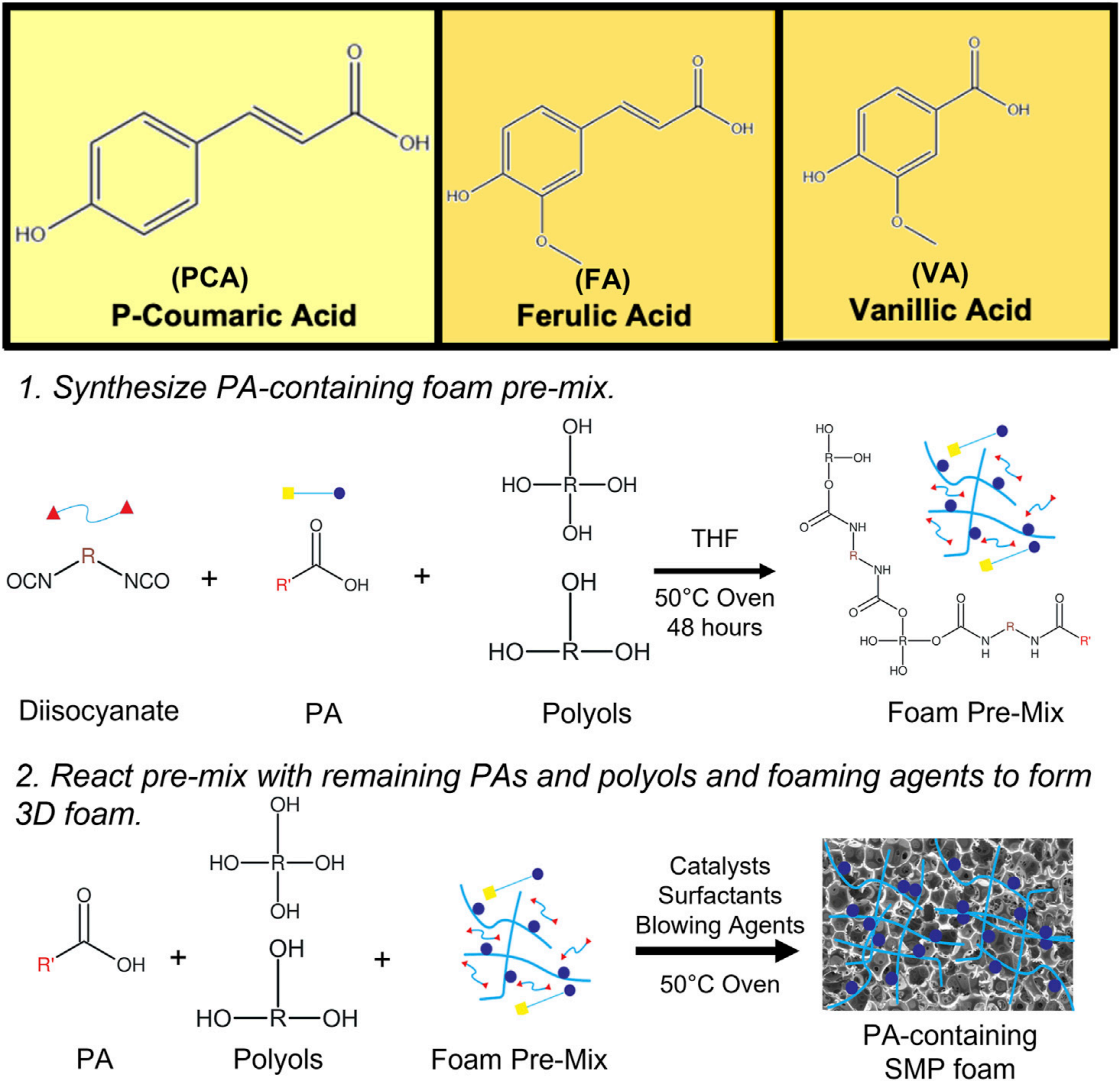
Chemical structures of PCA, VA, and FA and schematic representation of PA incorporation into SMP foams.

In our previous work, we characterized a library of PAs to analyze the effects of PA structures on antimicrobial and antioxidant activity. We showed that all tested PAs had antimicrobial activity and that PA antioxidant activity correlated positively with increased numbers of hydroxyl and/or methoxy groups on their rings. Additionally, we modified the carboxylic acid group of PAs with a polyurethane monomer analog and found that antimicrobial and antioxidant properties were maintained after modification. (Liu et al., 2020). Based on this study, we hypothesized that incorporation of PAs into polyurethanes by reaction of their carboxylic acid group with isocyanates could be used to impart antimicrobial and antioxidant properties to SMP foams. We identified p-coumaric acid (PCA), vanillic acid (VA), and ferulic acid (FA) as PAs with strong antimicrobial and antioxidant properties and selected them for incorporation into SMP foams, Figure 1. PA-containing SMP foams were synthesized according to a previously described method, Figure 1. (Monroe et al., 2018). The resulting foams were characterized in terms of surface chemistry, pore size and structure, thermal properties, and shape recovery profiles. We studied the antimicrobial efficacy of the foams against common wound pathogens. We also tested the ability of PA foams to scavenge hydrogen peroxide as an indication of their antioxidant properties, and we characterized cytocompatibility, hemolysis, and platelet interactions to study hemostatic material efficacy. These studies provide an initial indication of the ability to employ PA foams as hemostatic dressings that protect against infection in traumatic wounds.

## Materials and Methods

### Materials

All chemicals were purchased from Fisher Scientific (Waltham, MA, United States) unless otherwise specified. P-coumaric acid (PCA) and 1-ethyl-3-carbodiimide hydrochloride (EDC) were purchased from TCI America Inc. (Portland, United States); ferulic acid (FA), 4-dimethylaminopyridine (DMAP), and vanillic acid were purchased from Alfa Aesar (Haverhill, United States); T-131 and BL-22 were supplied by Evonik (Essen, Germany). Bacteria strains were purchased from ATCC (Manassas, VA, United States). All chemicals were used as received.

### Phenolic Acids-Containing Shape Memory Polymer Foam Synthesis

SMP foams were synthesized using a method adapted from Singhal et al. (Singhal et al., 2012). PAs were reacted at 50°C for 48 h with appropriate molar ratios of N,N,N′,N′-tetrakis (2-hydroxypropyl)ethylenediamine (HPED), triethanolamine (TEA), and excess hexamethylene diisocyanate (HDI) to form an isocyanate (NCO) pre-polymer with 42 mol% hydroxyl (OH) content. A hydroxyl (OH) solution was prepared with PAs, HPED, and TEA in tetrahydrofuran (THF) in an amount required to achieve a final 1:1 ratio of COOH (from PAs) + OH (from TEA and HPED):NCO. The OH solution was mixed with T-131 and BL-22 catalysts, EPH190 surfactant, and deionized water as a chemical blowing agent using a speed mixer (FlackTek, Inc., Landrum, SC). The OH solution was reacted with the NCO pre-polymer at 50°C to form a PA-containing SMP foam. Then, the foams were cooled to room temperature, cut into testing samples, and washed in 70% reagent alcohol (10 volume eq.). Foams were dried under vacuum overnight at room temperature. (Monroe et al., 2018). The synthesized foam formulations are shown in Table 1.

**TABLE 1:** Synthesized PA-containing SMP foam compositions. NCO: isocyanate, OH: hydroxyl.

Formulation	NCO equivalents (%)	OH equivalents (%)				Surfactant (%)	T-131 (%)	BL-22 (%)	Premix OH/NCO
	HDI	HPED	TEA	PA	Solvent				
Control foam	100	70	30	-	-	7	0.5	1.1	0.35
PCA foam	100	70	20	10	THF	9	0.5	1.1	0.38
VA foam	100	70	20	10	THF	11	0.8	1.1	0.35
FA foam	100	70	20	10	THF	9	0.50	1.1	0.35

PA incorporation into SMP foams was analyzed in terms of surface chemistry using attenuated total reflectance (ATR)-Fourier transform infrared (FTIR) spectroscopy (Nicolet i70 Spectrometer, Fisher Scientific, Waltham, MA) at 0.8 cm^−1^ resolution.

### Foam Density

SMP foam blocks (∼1 cm^3^, *n* = 3) were cut from the top, middle, bottom sections of synthesized foams. Each foam block was weighed using a gravimetric scale. The volumes were calculated from length, width, and height measurements obtained using a digital caliper. Sample density was calculated by dividing foam masses by their volumes.

### Pore Size and Structure

Samples (∼1 mm thick) were cut from the top, middle, and bottom sections of each foam parallel and perpendicular to the foaming direction and fixed to SEM sample holders with double-sided tape. Samples were coated with Au for 45 s using a sputter coater (Denton Vacuum Desk II, Moorestown, United States) and imaged using a Jeol NeoScope JCM-5000 Scanning Electron Microscope (SEM, Nikon Instruments, Inc., Melville, United States). Pore sizes were measured at the longest diameter of each visible pore using ImageJ software (NIH, Bethesda, United States).

### Foam Thermal Transitions

The glass transition temperature of dry and wet samples was measured using Q-200 differential scanning calorimeter (DSC, TA Instruments, Inc., New Castle, DE). The foam samples were cut into small pieces (3–5 mg). Dry foam samples (*n* = 3) were directly placed on a Tzero aluminum pan and covered with an aluminum lid (DSC Consumables, Inc., Austin, MN). Samples were subjected to the following program: 1) equilibrated at –40°C for 2 min, 2) heated to 120°C at 10°C min^−1^; 3) equilibrated at 120°C for 2 min, then cooled at −10°C min^−1^; 4) equilibrated at –40°C for 2 min again, and 5) heated to 120°C at 10°C min^−1^ in a second cycle.

The wet foam samples were immersed in 50°C DI water for ∼5 min and then pressed dry with Kim Wipes. Then, the wet samples (*n* = 3) were placed in a Tzero pan and covered with a lid. Samples were subjected to the following program: 1) equilibrated at –40°C for 2 min, 2) heated to 80°C at 10°C min^−1^. The Tg’s of dry and wet foam samples were collected from the second cycle and first cycle, respectively, via the inflection point of the DSC thermogram using TA instruments software (TA Instruments, Inc., New Castle, DE).

### Volume Recovery

Foam cylinders (*n* = 3, 4 mm diameter, 10 mm length) were heated to 100°C for 15 min, and the original volume of the samples was measured for each sample using a digital caliper. Then, the samples were placed in a crimper (Blockwise Engineering LLC, Temp, AZ) and manually crimped into a 2 mm diameter cylinder for 5 min at room temperature to complete the programming process. The samples were left for 24 h at room temperature to confirm shape fixity. The crimped foams were imaged, and their diameters were measured using Image J software (NIH, Bethesda, MD). A 0.5 mm diameter nickel-titanium wire (NDC, Fremont, United States) was threaded through the foam samples and used to suspend them in a 37°C water bath, where they were photographed every 10 s over 5 min. Foam dimensions in each image were measured and converted into volume measurements using Visual Studio software. The percent volume recovery of the sample was calculated using Eq. (1):

$$\%\;volume\;recovery=\left(\frac{recovered\;volume}{original\;volume}\right)\times 100$$

### Cell Viability

Cell viability was confirmed with mouse embryonic 3T3 fibroblast using an Alamar Blue assay. Cells were seeded in 24 well plates and incubated in Dulbecco’s Modified Eagle Medium (DMEM) with 10% fetal bovine serum (FBS) and 1% penicillin-streptomycin (PS) at 37°C overnight. The next day, foam samples (*n* = 3) were placed into Transwell inserts above the seeded cells and incubated for up to 24 h with fresh media. Empty inserts were used as the positive control group (*n* = 3), 30% H_2_O_2_ was added to the negative control group, and silver-based foam dressings (AREZA MEDICAL, Dallas, Tx) were used as a clinical control. After 3 and 24 h, the inserts with samples were removed from the well plate. Then, the media was removed, and 600 μL of 10% Alamar Blue was added. Cells were incubated for 2 h at 37°C. Then, 100 μL of the Alamar Blue solution was transferred into a 96 well plate from each well on the 24 well plate. A plate reader was used to analyze the fluorescence intensity with excitation of 530 nm and emission of 590 nm. Cell viability has measured as Eq. 2:

$$Cell\;Viaility\;\left(\%\right)=\frac{OD_{sample}}{OD_{positive\;control}}*100\%$$

where OD_sample_ is the optical density (O.D.) of sample, and OD_positive control_ is the O.D. of positive control (empty insert).

### Blood Interactions

For hemolysis measurements, samples were cut to have a uniform surface area of 143.8 cm^2^ (*n* = 6). XStat and QuikClot were used as clinical controls, the negative (non-hemolytic) control was PBS, while the positive (hemolytic) control was DI Water. Each sample was incubated in 1X PBS for 5 h at 37°C in 15 ml centrifuge tubes, and then 5 ml of fresh PBS was added prior to testing. The controls included empty tubes with of 5 ml PBS or DI water. After incubation, 5 ml of anticoagulated whole porcine blood (Lampire Biological Laboratories, Everett, PA) was warmed to room temperature, and mixed by inversion. Blood (0.1 ml) was added to each tube and incubated at 37°C for 1 h. Then, samples were removed from tubes, and tubes were centrifuged at 1000 RPM for 10 min. After centrifuging, 200 µL of supernatant was transferred from each tube into a 96 well-plate to be read on a microplate reader (absorbance at 545 nm).

For platelet interactions, each sample was cut to a volume of 0.5 cm^3^ and placed into a 24 well-plate. Whole blood (2 ml) was added to each sample, and they were incubated for 1 h at room temperature. Then, samples were rinsed with PBS to wash away non-attached platelets. Glutaraldehyde (1 ml of 2.5%) was added to each sample and incubated overnight at 4°C in the refrigerator to fix platelets. Samples were dehydrated in increasing concentrations of ethanol for 30 min each, going from 50%, to 70%, to 95%, and then to 100%. Samples were then vacuum dried overnight at room temperature, and once dry were imaged via SEM to visualize platelet interactions.

### Hydrogen Peroxide Scavenging

To quantify antioxidant properties, PA foams were cut into cylinders via biopsy punch (Sklar Instruments, West Chester, PA) (8 mm diameter, 2 mm length, *n* = 3). A 0.002% H_2_O_2_ solution, 10x dying agent solution (0.1 mg/ml horse radish peroxidase (HRP) and 0.2 mg/ml phenol red dye (PRD)), and 1 M NaOH solution were prepared in deionized water. Samples were placed into a 96 well plate, and 100 μL of PBS was added. Blank solutions (100 μL PBS) were added to empty wells on the plate. The plate was placed on a shaker table for 1 h, and then, 10 μL of 0.002% H_2_O_2_ solution was added to each well. After 10 min incubation in a shaker table at 37°C, 100 μL of the 10x HRP-PRD solution was added for 15 min in a shaker table at 37°C. Subsequently, 5 μL of 1 M NaOH was added to each sample well, and 100 μL of solution was transferred from each sample well into empty wells. Hydrogen peroxide scavenging was analyzed immediately using a plate reader (FLx800, Bio-Tek Instrument, Inc.) at an absorbance of 610 nm. The antioxidant properties were quantified in terms of hydrogen peroxide scavenging activity (H_s_) using Eq. 3:

$$H_{s}=100\%\times \left[C_{0}-\left(C_{foam}-C_{0}\right)\right]$$

where C_0_ is the absorbance value of the blank group with PBS only and C_foam_ is the absorbance value of the PA foams.

### Antimicrobial Properties

SMP foams were cut into cylinders via biopsy punch (6 mm diameter, 5 mm length, *n* = 3). The samples were sterilized by immersion in 70% ethanol for 30 min and then washed in sterile phosphate buffered saline (PBS) 3 times. Silver-based foam dressings (AREZA MEDICAL, Dallas, Tx) served as a clinical control. *Escherichia Coli (E. coli), Staphylococcus aureus (S. aureus),* or *Staphylococcus epidermidis (S. epidermidis)* were grown in 5 ml of sterile lysogeny broth (LB) at 37°C. After 16 h, 1 ml was taken from the overnight bacterial solution, diluted into 10 ml of LB, and incubated at 37°C until the culture reached an O.D. of 0.6. O.D. was confirmed using a plate reader (FLx800, Bio-Tek Instrument, Inc.). Samples were placed into a sterile 96 well plate, and 100 μL of bacterial solution was added onto each sample. The well plate was placed in a shaker table to culture at 37°C for 1 h. The bacterial solutions were diluted by 10^7^ in sterile LB, and 10 μL of the diluted bacterial solution was taken from each sample and pipetted onto a LB-agar plate to culture for 18 h at 37°C. Images were obtained of each drop area, and colony forming units (CFUs) were measured by counting the number of colonies.

### Statistics

All statistical analysis was done using GraphPad Prism. Data are reported as mean ± standard deviation. Student’s T-tests were performed to determine differences between PA foams and controls. Statistical significance was taken as *p* < 0.05.

## Results

### Synthesis of Phenolic Acids-Containing Shape Memory Polymer Foams

**FIGURE 2:**
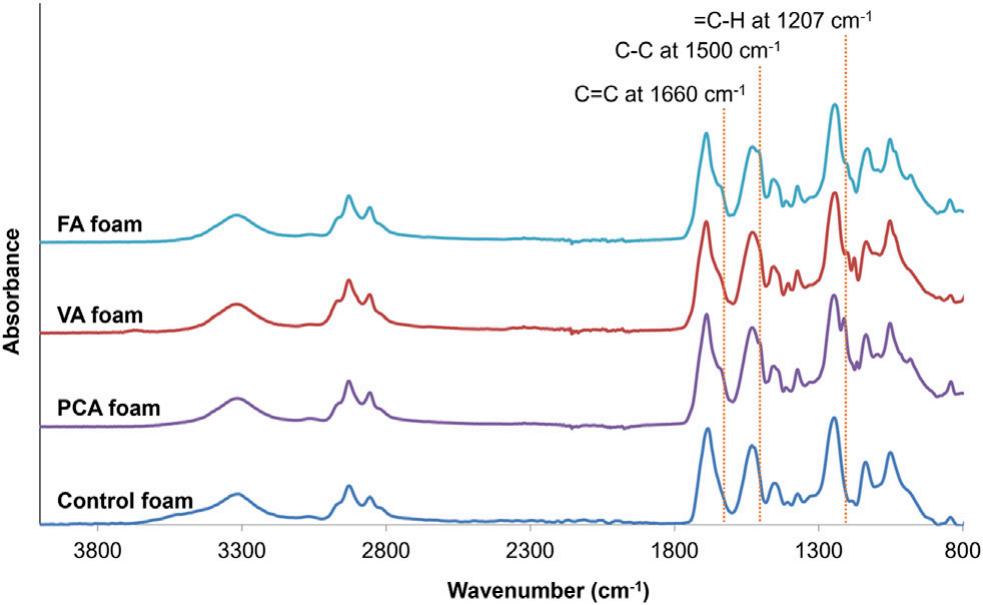
FTIR spectra of synthesized PCA, VA, FA, and control foams. Incorporation of PCA, FA, and VA into SMP foams resulted in the addition of a C=C peak at ∼1,660 cm^−1^, a C-C peak at ∼1,500 cm^−1^, and a = C-H peak at ∼1,207 cm^−1^, corresponding to the phenolic ring of the PAs. These key peaks are highlighted with dotted lines.

In Figure 1, the schematic shows a simple and effective way to directly incorporate PAs into polyurethane foams. The functional carboxylic acid group of PAs is reacted with an isocyanate group on hexamethylene diisocyanate (HDI) to form an amide linkage. Successful incorporation of PAs into the SMP foam was confirmed by surface chemistry analysis using FTIR spectroscopy, Figure 2. All foams show evidence of urethane formation (NH peak at ∼3,300 cm^−1^ and C=O of urethane peak at ∼1,680 cm^−1^). Incorporation of PCA, FA, and VA into SMP foams was evidenced by the addition of a C=C peak at ∼1,660 cm^−1^, a C-C peak at ∼1,500 cm^−1^, and a = C-H peak at ∼1,207 cm^−1^, corresponding to the phenolic ring of the PAs. This result indicates that we successfully incorporated PAs into the SMP foam network.

### Structural Properties

**FIGURE 3:**
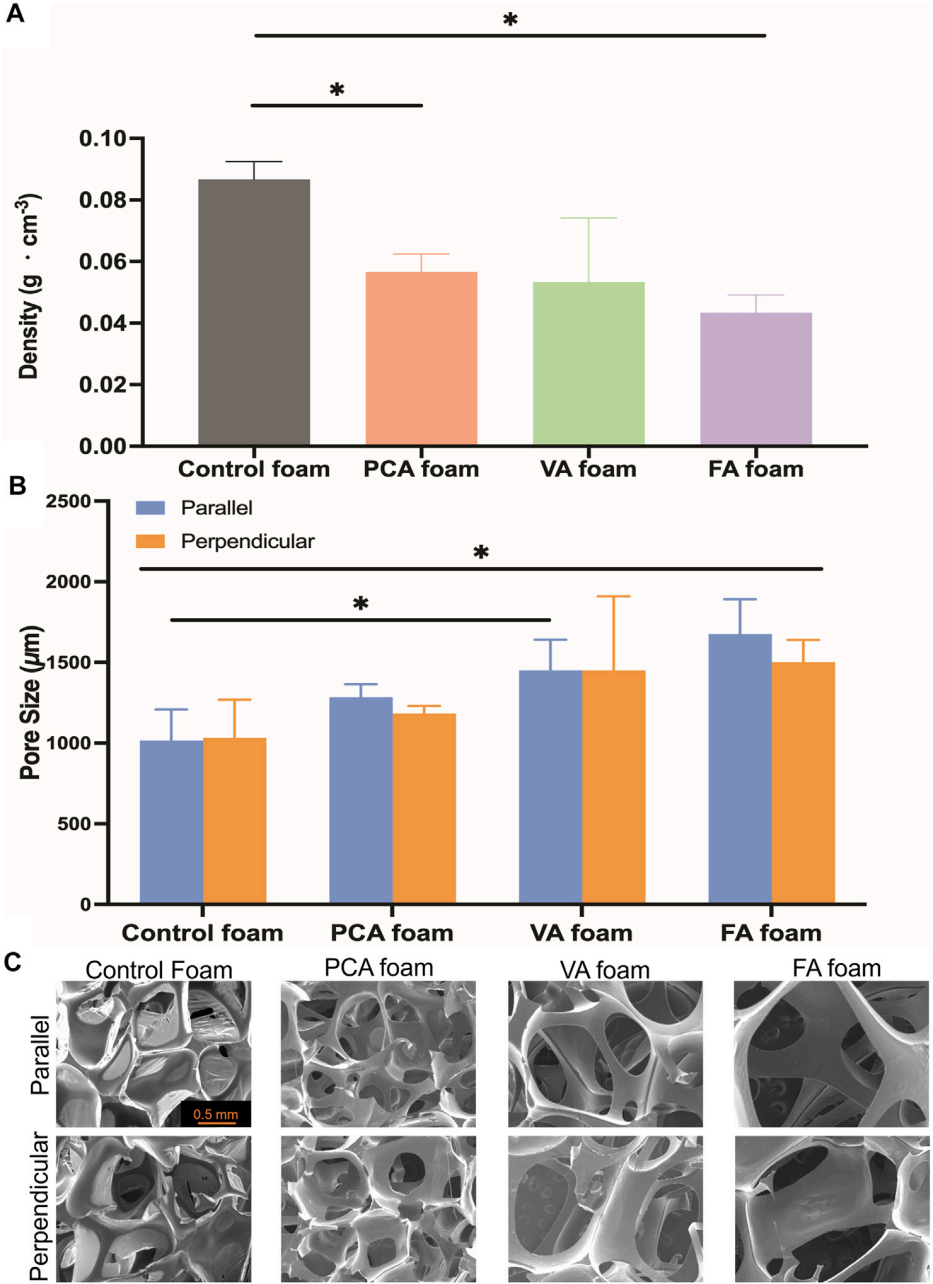
Structural properties of PA-containing SMP foams. **(A)** Density; **(B)** Pore size (parallel and perpendicular to the foaming direction); **(C)** Scanning electron micrographs. Scale bar of 0.5 mm applies to all images. *n* = 3, mean ± standard deviation displayed, *p < 0.05.

Foam density and pore sizes were quantified to assess the success of foam blowing during the incorporation of PAs into SMP foams. The PA foams are less dense than control foams, Figure 3A, and PA foams have consistent pore sizes between 1,000 and 1,500 μm, with larger pores compared with the ∼1,000 µm control SMP foam, Figure 3B. The morphology of the pores in PA foams are uniform and rounded with high interconnectivity, Figure 3C.

### Thermal and Shape Memory Properties

**FIGURE 4:**
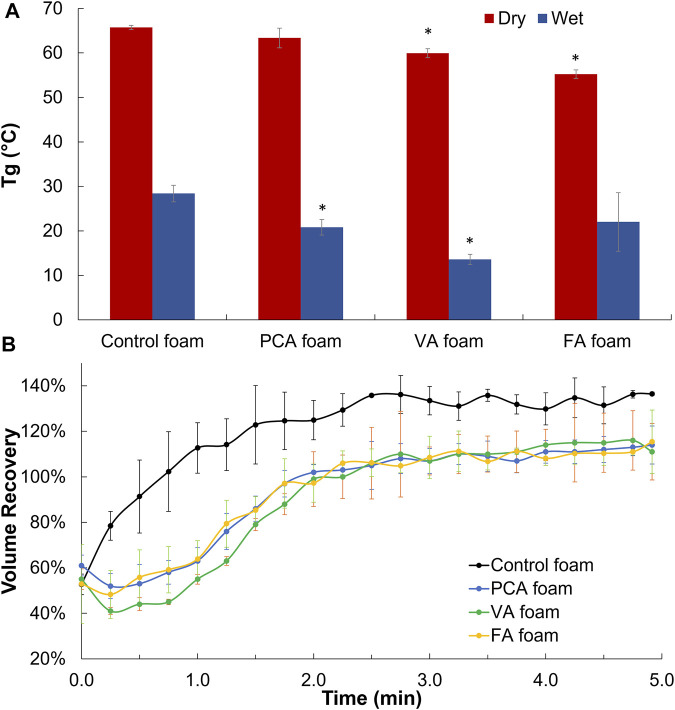
Thermal and shape memory properties of PA-containing SMP foams. **(A)** Dry and wet glass temperature (*T*g). **(B)** Volume recovery profile in body temperature water. *n* = 3, mean ± standard deviation displayed, *p < 0.05 relative to control foam in same condition.

The glass transition temperatures (T_g_) of PA-containing SMP foams were measured under dry and wet conditions. Ideally, dry T_g_ is above 45°C to enable stable storage of SMP foams in the secondary shape, and wet T_g_ is below 37°C (body temperature) to enable expansion back to the primary shape after implantation. In general, T_g_’s were reduced after PA incorporation, but all foams still met these success criteria, Figure 4A. To assess shape memory properties, compressed PA SMP foams were placed in 37°C water to measure volume expansion back to their original, expanded shape, Figure 4B. All PA foams achieved 100% volume expansion within ∼2 min.

### Cytocompatibility

**FIGURE 5:**
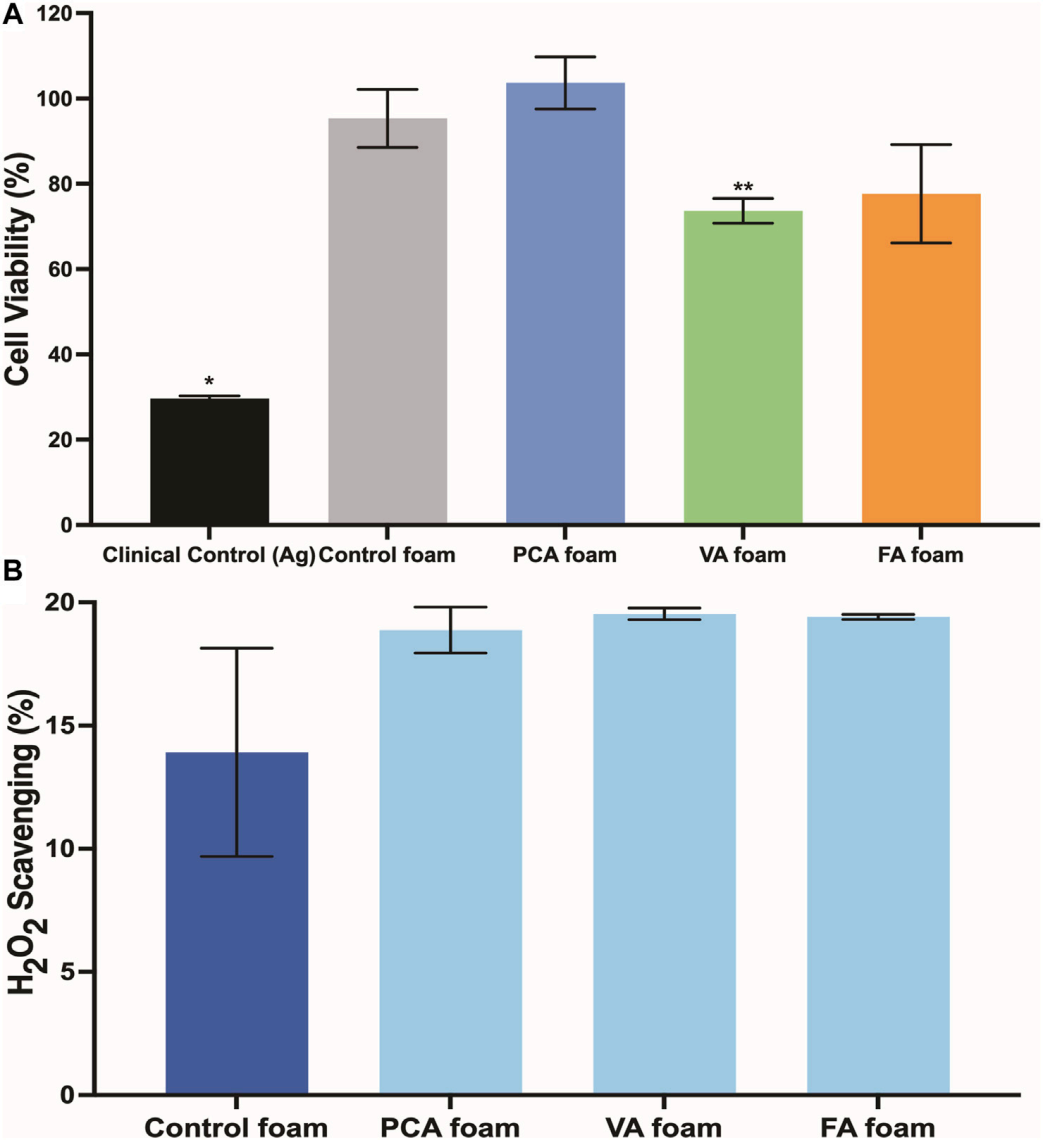
**(A)** 3T3 fibroblast viability in the presence of PA foams at 24 h *n* = 3, mean ± standard deviation displayed, *p < 0.05 relative to all other samples; **p < 0.05 relative to control foam. **(B)** Antioxidant properties of PA-containing SMP foams as quantified by hydrogen peroxide scavenging. *n* = 3, mean ± standard deviation displayed.

The viability of 3T3 fibroblasts was characterized over 24 h of incubation with SMP foams, Figure 5A. Cell viability of >79% was maintained for all samples over 24 h, with all but VA foams having comparable viability to control foams and statistically higher viability than the clinical silver-based control.

### Blood Interactions

**FIGURE 6:**
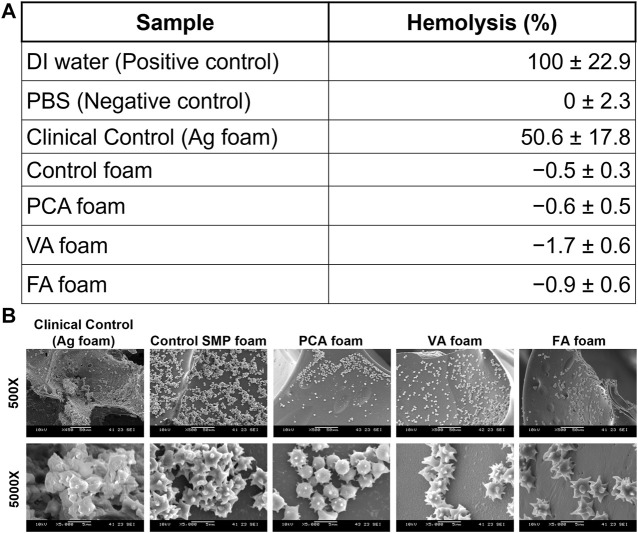
**(A)** Hemolytic properties of PA-containing SMP foams. n = 3, mean ± standard deviation displayed. **(B)** SEM images of platelet attachment to foams.

In Figure 6A, it can be seen that all SMP foam samples induce essentially 0% hemolysis. The positive control with DI water results in 100% hemolysis due to the hypotonic effect on red blood cells. (Goodhead and MacMillan 2017). Hemolysis below 2% is generally accepted as nonhemolytic, showing the PA-containing SMP foams exhibit very low hemolytic potential. (Totea et al., 2014). In contrast, the AREZA^®^ silver foam dressing induced 51% hemolysis, which is higher than the ISO 10993 standard of 5% for blood-contacting materials. (ISO 2009). In Figure 6B, platelet attachment can be observed on all foam surfaces. Spiked protrusions on the surfaces of platelets indicate activation, which is an important precursor to clotting. Overall platelet density on PA-containing SMP foams was qualitatively lower than that on the controls.

### Antimicrobial and Antioxidant Properties

**FIGURE 7:**
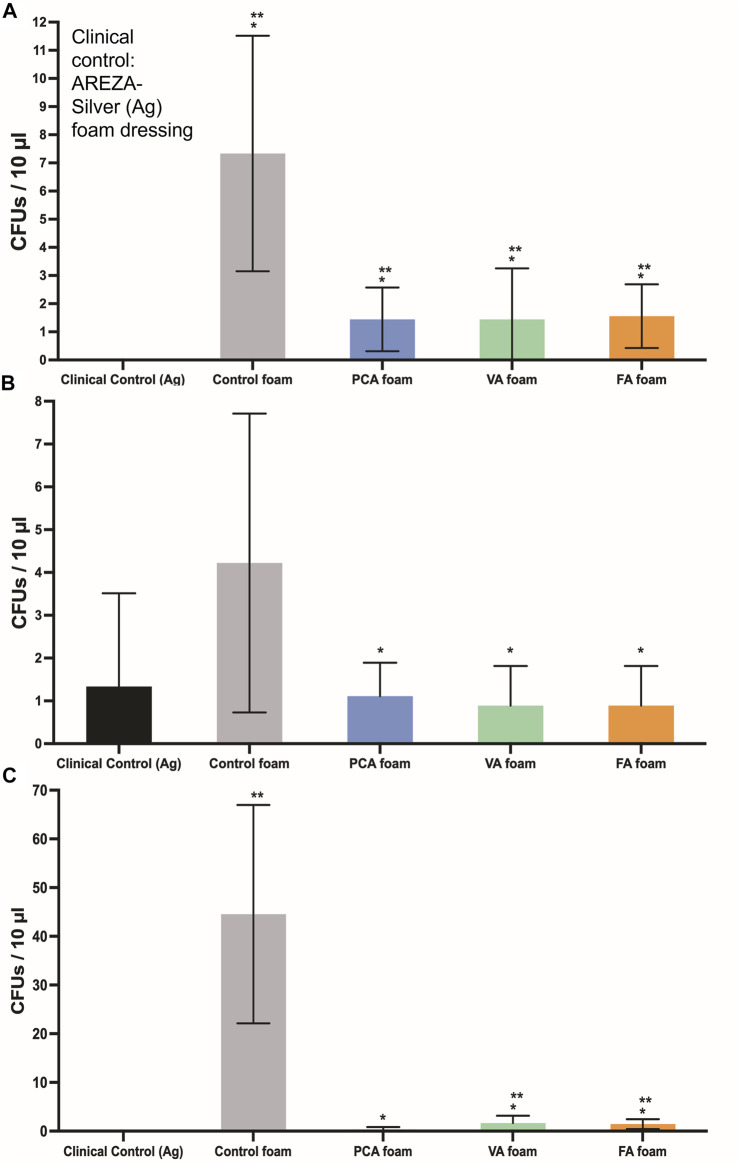
CFU densities of bacteria after incubation with SMP foams. **(A)** *E. coli,* **(B)** *S. aureus*, and **(C)** *S. epidermidis*. Clinical control: AREZA Medical-silver based foam dressings. *n* = 9, mean ± standard deviation displayed, *p < 0.05 relative to the control foam; **p < 0.05 relative to the clinical control.

The PA-containing SMP foams show a general increase in hydrogen peroxide scavenging relative to control foams, Figure 5B, which demonstrates that some of the antioxidant properties of PAs are retained after incorporation into the SMP foams. To quantify the antimicrobial properties of PA foams, the colony forming unit (CFU) densities of *E. coli* (gram-negative)*, S. aureus* (gram-positive)*,* and *S. epidermidis* (gram-positive) wer quantified after incubation of bacteria with foams. In Figure 7A, it can be seen that PA-containing SMP foams reduce *E. coli* growth in comparison with control foams. The silver-based foam that was used as a clinical control shows superior antimicrobial efficacy against *E. coli* in comparison with PA-containing foams. PA foams also showed improved antimicrobial properties against *S. aureus* and *S. epidermidis* relative to control foams (Figures 7B,C). All PA foams were comparable to clinical control in studies with *S. aureus*, and PCA foams exhibited comparable antimicrobial ability to the clinical control against *S. epidermidis*.

## Discussion

After confirming successful incorporation of PAs into SMP foams, their properties were measured relative to controls. The reduced density and interconnected pores of PA-containing SMP foams may be due to the use of solvent tetrahydrofuran (THF) that was required for PA solubilization during foaming, which could serve as a physical blowing agent that vaporizes during foaming to form new pores and reduce overall density. In addition, the reaction between the carboxylic acid group of PAs and the isocyanate group of HDI produces carbon dioxide, which provides an additional blowing agent for the PA foams. It is also possible that PAs serve as a pore opener, with hydrophilic groups on their rings that increase interactions between the polymer and the water chemical blowing agent.

In general, the pore size and structures correlate well with the density results (i.e., pore size increases resulted in lower density). While PA foam pores were statically similar, FA and VA foams had the largest, most open pore structure. This correlates with the addition of the methoxy group on the ring structures in these PAs. This methoxy group in addition to the pendent hydroxyl groups on FA and VA may have aided in pore opening to increase pore size and interconnectivity in comparison with PCA, which has a single pendant hydroxyl group. The interconnected pores that result from PA incorporation could increase blood permeability in the foams and/or allow for cell migration into the foam scaffold during healing. (Tan et al., 2010). Overall, these studies show that pore size can be tuned during foam synthesis, PA incorporation did not negatively affect the foaming process, and PA incorporation provides a new way to increase pore interconnectivity in SMP foams.

The thermal properties of PA-containing foams would enable their use as hemostatic dressings. Namely, the dry T_g_ values of all PA foams are greater than 55°C; thus, they can theoretically maintain their compressed shape under dry storage conditions up to 55°C without undergoing premature expansion. The wet T_g_ values of all PA foams are less than 30°C, which ensures that they can expand when exposed to water in body temperature blood, even in cases of hypothermia. This may be an important consideration, as 18% of 2,848 patients in combat support hospitals in Iraq had hypothermia with body temperatures below 36°C. (Arthurs et al., 2006).

Incorporation of the monofunctional PA group reduces the overall foam crosslink density, thereby decreasing T_g_; however, we hypothesize that the stiff phenolic ring of PAs reduces the flexibility of the SMP backbone, thereby minimizing T_g_ variations. (Singhal et al., 2013). The slight decrease in dry T_g_ of FA and VA foams correlates with the flexible, pendant methoxy group, which may increase the overall flexibility of the polymer backbone to drive a small T_g_ reduction. The rapid volume expansion of PA-containing SMP foams indicates their potential for filling wounds after application and exposure to body temperature blood. No observable trends were measured between different PAs and volume expansion rates, indicating that their minor effects on T_g_ do not alter shape memory properties. Overall, the incorporation of PAs did not negatively affect the thermal or shape memory properties of polyurethane SMP foams.

Notably, all SMP foams show statistically higher cytocompatibility than clinical silver-based foam. Thus, these materials would likely have fewer detrimental effects on healing than those that have been previously observed with silver-based antimicrobial biomaterials. (Atiyeh et al., 2007; Chopra 2007; Khansa et al., 2019). For clinical use of hemostatic dressings, blood-contacting biomaterials should interact favorably with the blood components. Hemocompatibility is an important criteria for testing blood-contacting medical devices, according to ISO 10993–4. (Weber et al., 2018). Hemocompatibility testing involves characterization of coagulation, including platelet interactions (Liu et al., 2014), and hemolysis. The increased hemolysis that was observed in the presence of the silver-based clinical control indicates that it is not an ideal blood-contacting material, due to its potential for damaging red blood cells. This result further demonstrates the potential benefits of employing natural antimicrobial species in biomaterial scaffolds.

Our previous *in vivo* work in a porcine liver injury model demonstrates that control SMP foams rapidly stabilize hemorrhages, resulting in reduced mortality in comparison with clinical controls. (Beaman 2021). While coagulation is a complex process with many potential drivers, analysis of platelet interactions provides a strong initial characterization of a biomaterial’s ability to promote blood clotting, as platelet attachment is mediated by prior plasma protein adsorption and platelet activation is followed by thrombus formation. (Xu et al., 2014; Hanson et al., 2020). Despite previous reports on the procoagulant properties of some PAs, platelet attachment density was qualitatively lower on PA-containing foams. (Huang et al., 2010; Luo et al., 2017). However, the clear activation of platelets on PA foam surfaces is a promising indication that blood clotting via thrombus formation could be achieved in these materials. Future studies will focus on measuring coagulation times, quantifying platelet attachment numbers, and measuring dynamic blood/material interactions under flow to better understand the potential effects of PA incorporation on SMP foam clotting. If blood clotting profiles are dramatically altered in PA foams, these biomaterials still serve as potentially valuable wound dressings with reduced infection risks and high cell and blood compatibility.

Upon confirming that desired architectural, thermal, and biological properties were maintained, the function of PAs in the foams was characterized. The antimicrobial tests show that PA-containing SMP foams are effective against a range of common wound pathogens, and in some cases are as effective as the silver-based clinical control. This is important, since PA foams also have improved cytocompatibility and hemocompatibility relative to the silver-based foam. Previous studies have shown that PA antimicrobial activity increases with decreased numbers of ring side groups. (Liu et al., 2020). While there were minimal differences in antimicrobial activity in these studies, a general trend of increased efficacy with PCA foams that only have a single pendant hydroxyl was observed. Thus, it may be beneficial to focus on PAs with lower numbers of side groups (such as PCA) in future work on antimicrobial PA-containing scaffolds.

PA foams offer the potential to scavenge radical species in the wounds and thus promote wound healing; however, antioxidant activity was modest in these studies. The similarities between the scavenging activities of the three different PA foams was expected, based on our previous work that showed that PCA, FA, and VA have comparably high antioxidant activity. In future work, increased antioxidant properties will be pursued by adding in physically-incorporated PAs to the foams. Additionally, we will explore more complex antioxidant interactions using cellular-based antioxidant assays. (Wolfe and Liu 2007). In summary, these results demonstrate successful synthesis of PA-containing SMP foams that maintain the antimicrobial functionality of PAs after incorporation and provide some potential protection against ROS, which could have clinical benefits in traumatic wound healing.

## Conclusion

To address traumatic wounds, such as gunshot wounds, the most commonly used current treatment option is a combination of gauze and tourniquets. These dressings are insufficient for non-compressible wounds and do not provide infection protection, resulting in high morbidity and mortality. To address this clinical need, we developed a PA-based SMP foam with antimicrobial and antioxidant properties. The shape memory properties enable easy application to deep and/or irregularly-shaped wounds, while the PAs impart antimicrobial efficacy against a range of common wound pathogens (*E. coli*, *S. aureus*, and *S. epidermidis*) and antioxidant activity. In addition, the excellent hemocompatibility and cytocompatibility of PA-containing SMP foams provide a potential candidate for use as an antimicrobial hemostatic wound dressing.

## Data Availability

The raw data supporting the conclusions of this article will be made available by the authors, without undue reservation.
